# Supplementary material for: Structural basis for receptor selectivity and inverse agonism in S1P5 receptors
Source: Nat Commun. 2022 Aug 12;13:4736. doi: 10.1038/s41467-022-32447-1 (PMC9374744; doi:10.1038/s41467-022-32447-1)
Supplement: Supplementary file 1 — Supplementary Information [file 41467_2022_32447_MOESM1_ESM.pdf]

Supplementary Information

Structural basis for receptor selectivity and inverse agonism in $S1P_5$ receptors

Elizaveta Lyapina*[1], Egor Marin*[1,2], Anastasiia Gusach*[1,3], Philipp Orekhov[1,4,5], Andrey Gerasimov[6], Aleksandra Luginina[1], Daniil Vakhrameev[1], Margarita Ergasheva[1], Margarita Kovaleva[1], Georgii Khusainov[1,7], Polina Khorn[1], Mikhail Shevtsov[1], Kirill Kovalev[1,8], Sergey Bukhdruker[1], Ivan Okhrimenko[1], Petr Popov[1,9], Hao Hu[10], Uwe Weierstall[10], Wei Liu[11], Yunje Cho[12], Ivan Gushchin[1], Andrey Rogachev[1,13], Gleb Bourenkov[8], Sehan Park[14], Gisu Park[14], Hyo Jung Hyun[14], Jaehyun Park[14,15], Valentin Gordeliy[16], Valentin Borshchevskiy[1,13#], Alexey Mishin[1#], Vadim Cherezov[17#].

[1]Research Center for Molecular Mechanisms of Aging and Age-related Diseases, Moscow Institute of Physics and Technology, Dolgoprudny 141701, Russia

[2]Groningen Biomolecular Sciences and Biotechnology Institute, University of Groningen, Nijenborgh 4, 9747 AG Groningen, The Netherlands

[3]Present address: MRC Laboratory of Molecular Biology, Cambridge, CB2 0QH, UK

[4]Faculty of Biology, Lomonosov Moscow State University, Moscow 119991, Russia

[5]Faculty of Biology, Shenzhen MSU-BIT University, Shenzhen 518172, China

[6]Vyatka State University, Kirov 610020, Russia

[7]Present address: Division of Biology and Chemistry, Paul Scherrer Institute, Forschungsstrasse 111, 5232 Villigen PSI, Switzerland

[8]European Molecular Biology Laboratory, Hamburg unit c/o DESY, Hamburg, Germany

[9]Present address: iMolecule, Skolkovo Institute of Science and Technology, Bolshoy Boulevard 30, bld. 1, Moscow 121205, Russia

[10]Department of Physics, Arizona State University, Tempe, AZ 85281, USA

[11]Cancer Center and Department of Pharmacology and Toxicology, Medical College of Wisconsin, Milwaukee, WI 53226, USA

[12]Department of Life Science, Pohang University of Science and Technology, Pohang, Republic of Korea

[13]Joint Institute for Nuclear Research, Dubna 141980, Russia

[14]Pohang Accelerator Laboratory, POSTECH, Pohang 37673, Republic of Korea

[15]Department of Chemical Engineering, POSTECH, Pohang 37673, Republic of Korea

[16]Institut de Biologie Structurale (IBS), Université Grenoble Alpes, CEA, CNRS, Grenoble 38400, France

[17]Bridge Institute, Department of Chemistry, University of Southern California, Los Angeles, CA 90089, USA

[*]These authors contributed equally: Elizaveta Lyapina, Anastasiia Gusach, Egor Marin.

[#]Correspondence and request for materials should be addressed to V.C. (email: cherezov@usc.edu), A.M. (email: mishinalexey@phystech.edu) or V.B. (email: borshchevskiy.vi@phystech.edu).

Supplementary Figures

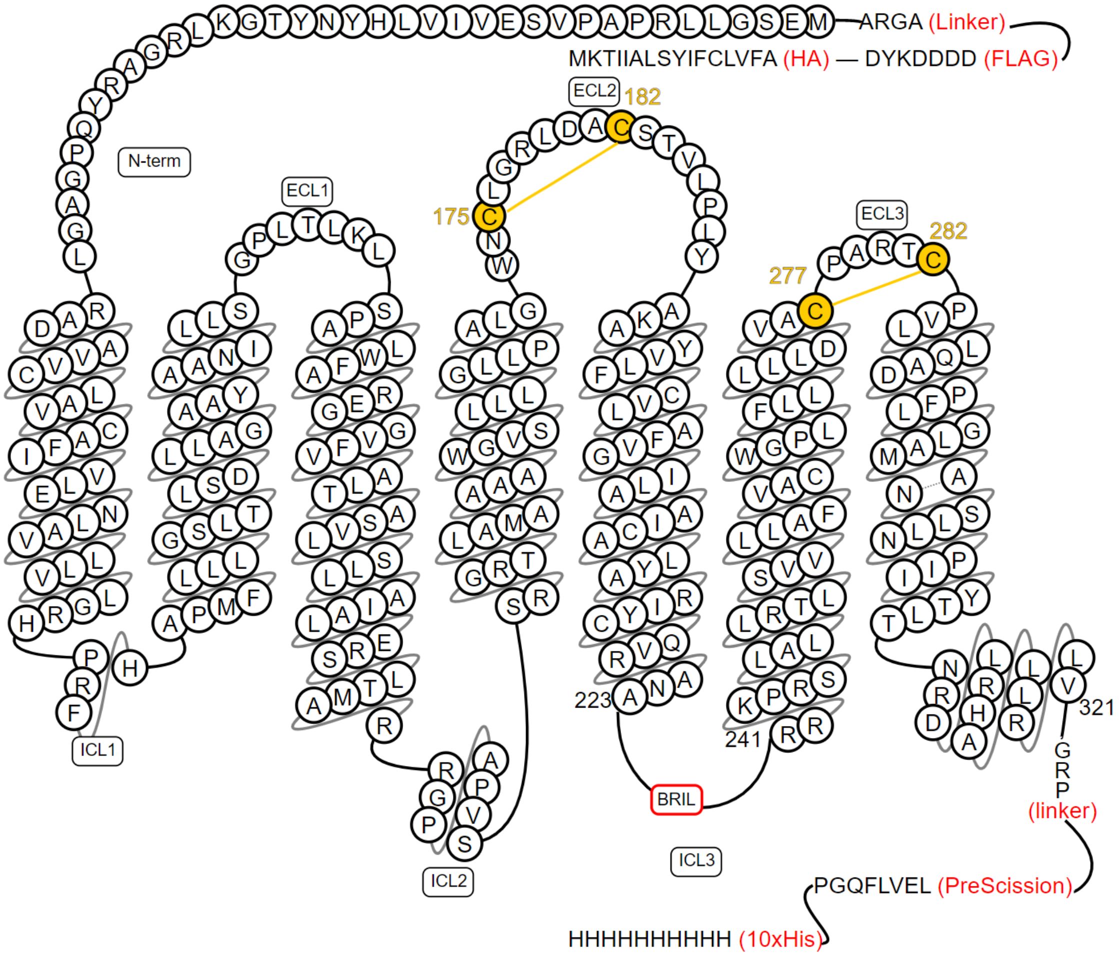

Supplementary Fig. 1 Crystallization construct of S1P$_5$. HA signal peptide, FLAG tag, and AGRA linker are attached to the N-terminus; BRIL is inserted in the ICL3 between residues A223 and R241; the C-terminus is truncated at V321 followed by GRP linker, PreScission site, and decahistidine tag.

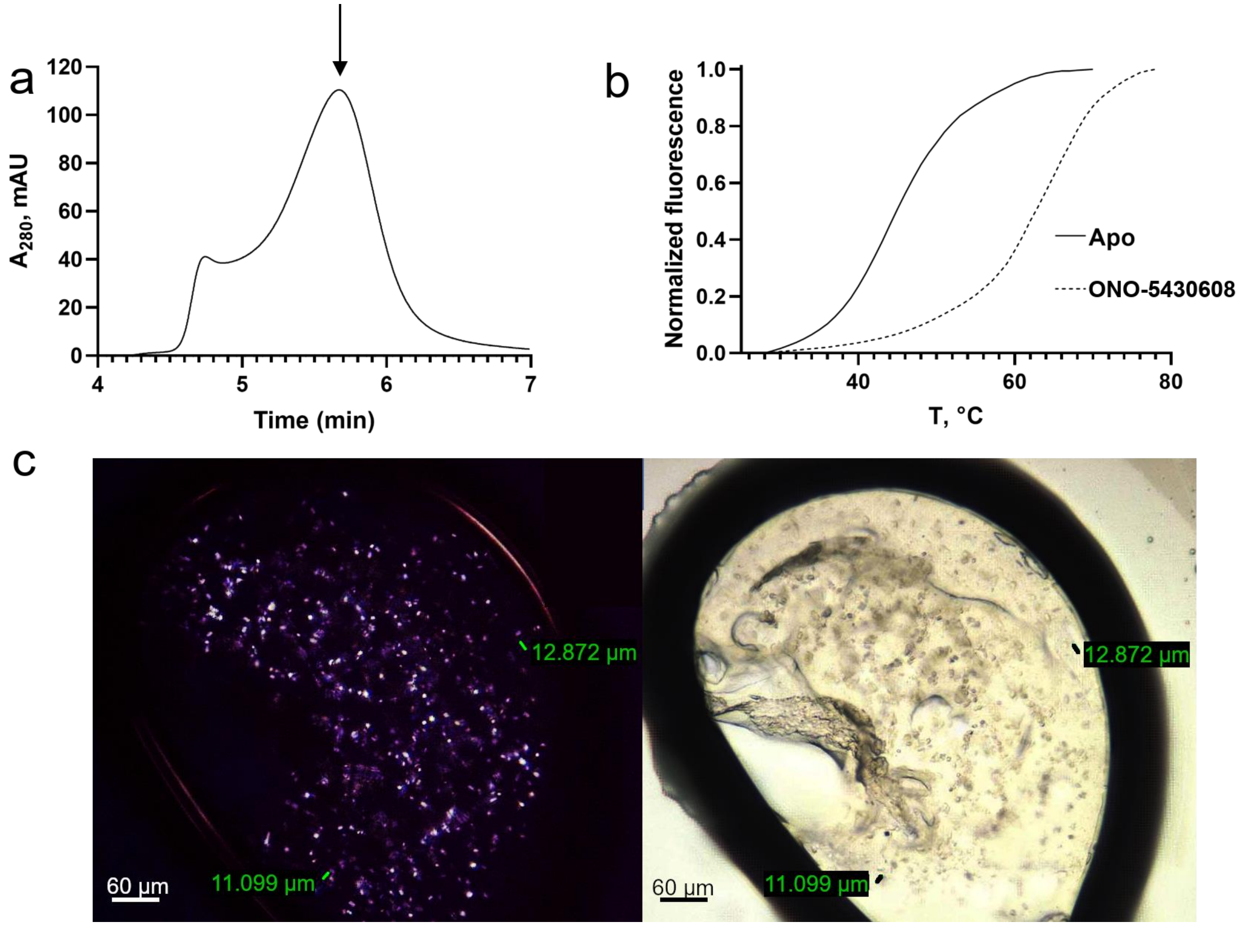

Supplementary Fig. 2 Characterization and crystallization of S1P$_5$-ONO-5430608. a Analytical size exclusion chromatography analysis of purified S1P$_5$ in complex with ONO-5430608, showing mostly monomeric protein preparation (the monomer peak is shown with an arrow). **b** Thermal shift assay using CPM fluorescence. ONO-5430608 increases the thermal stability of S1P$_5$ by 19 °C. **c** Crystals of S1P$_5$-ONO-5430608 grown in lipidic cubic phase as visualized by cross-polarized and direct light microscopy. Crystallization trials have been repeated 4 times yielding consistently diffracting crystals.

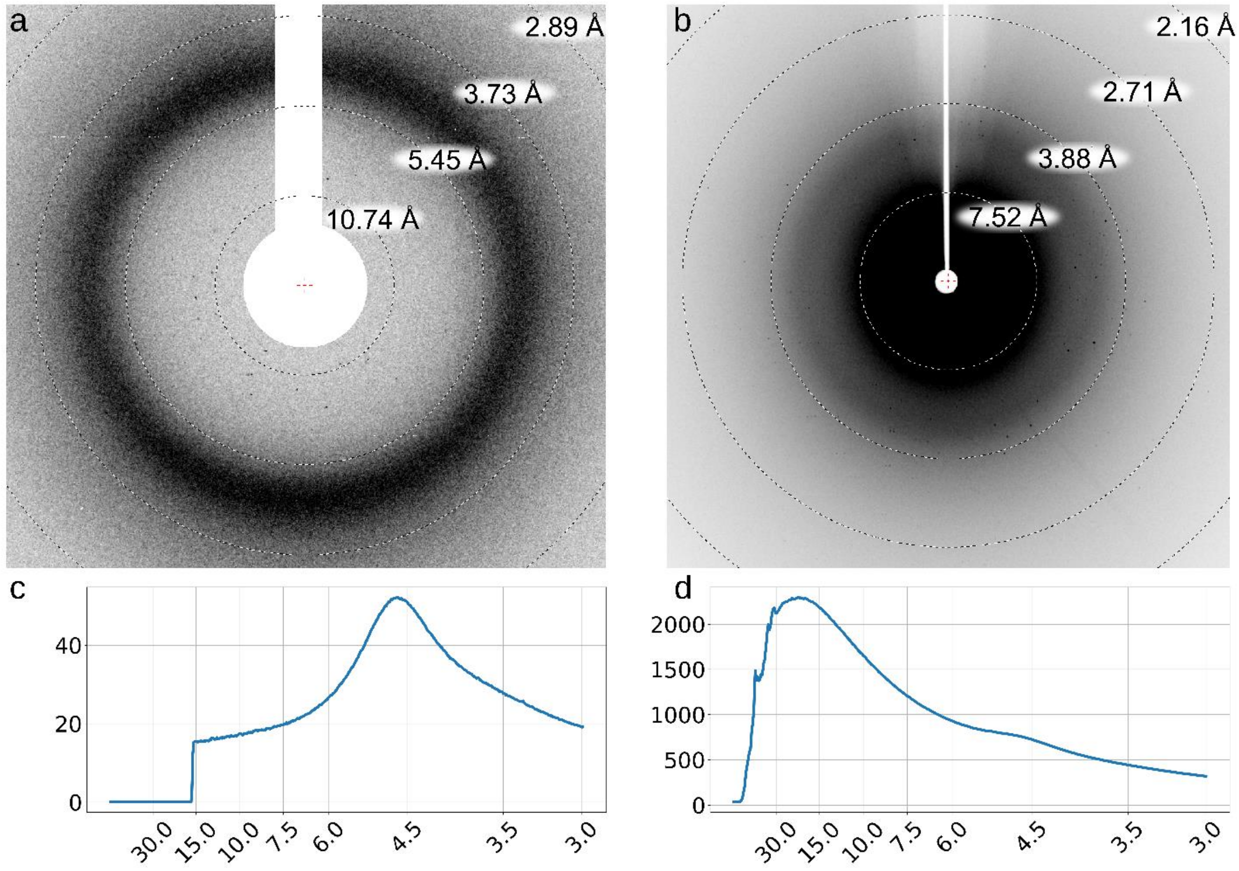

Supplementary Fig. 3 Examples of diffraction images collected at PAL-XFEL. a, b Diffraction images. **c, d** radial profiles. Diffraction image (**a**) and its radial profile (**c**) correspond to a typical low background experiment. Diffraction image (**b**) and its radial profile (**d**) correspond to the high background data collection described in this article.

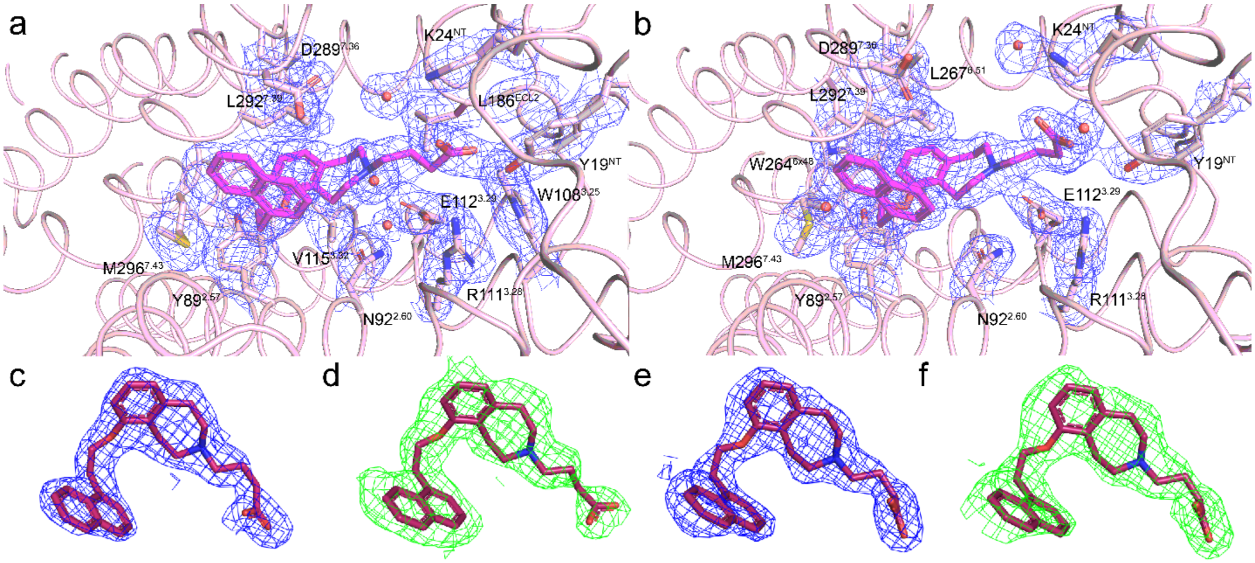

Supplementary Fig. 4 Examples of electron density. a, b 2*m*Fo-*D*Fc electron density maps around ONO-5430608 and ligand binding pocket residues and water molecules within 4 Å of the ligand in chains A (**a**) and B (**b**), contoured at 1.0 σ level. **c, e** 2*m*Fo-*D*Fc electron density maps around ONO-5430608 in chains A (**c**) and B (**e**), contoured at 1.5 σ. **d, f** Simulated annealing ligand omit 2*m*Fo-*D*Fc electron density maps around ONO-5430608 in chains A (**d**) and B (**f**), contoured at 1.5 σ.

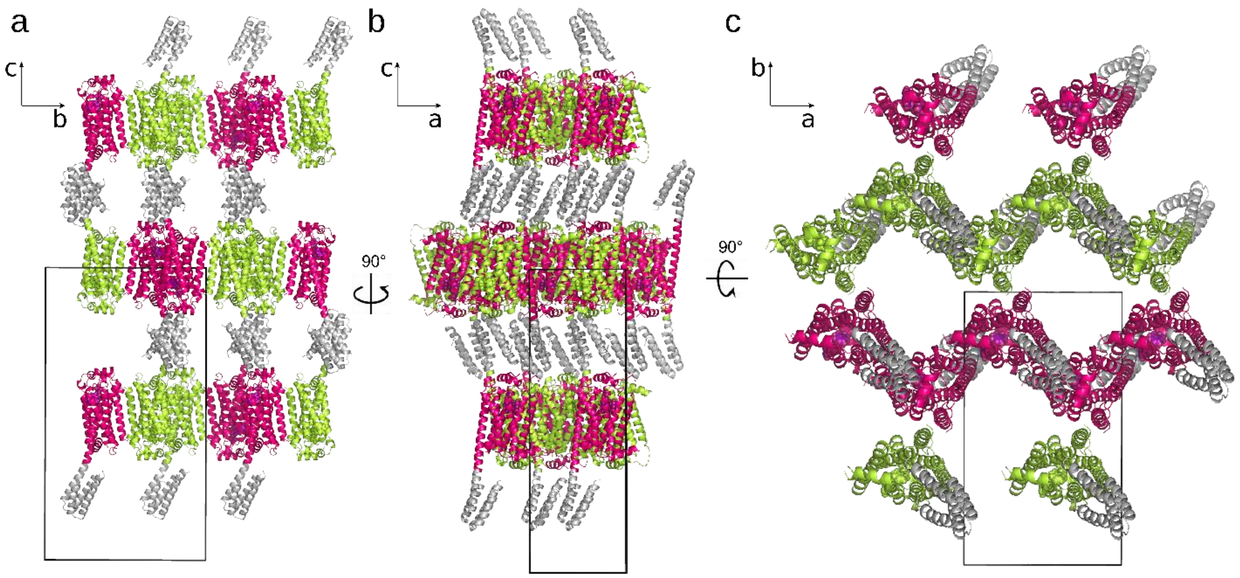

Supplementary Fig. 5 Crystal packing of S1P$_5$-ONO-5430608. a-c Crystal packing is shown in three orthogonal orientations. The unit cell is outlined by a black box. S1P$_5$ is shown in pink (chain A) and light green (chain B), BRIL fusion partner - in grey, ligand - in purple.

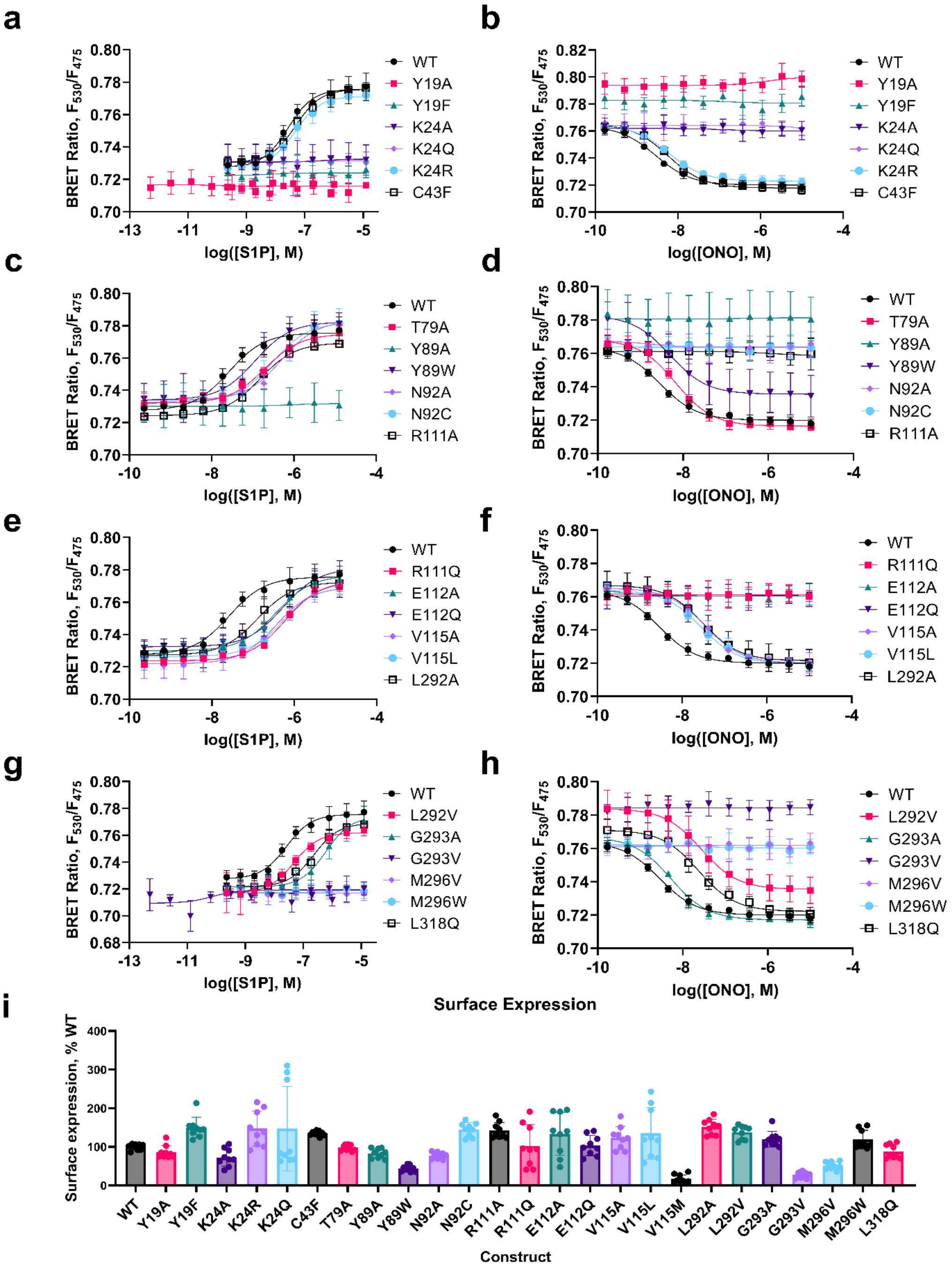

Supplementary Fig. 6 Dose-response curves for S1P and ONO-5430608 and cell surface expression for WT and mutants of S1P$_5$. a, c, e, g S1P- stimulated cAMP reduction at WT S1P$_5$ and mutants, measured by BRET-based EPAC sensor. **b, d, f, h** Inhibition of forskolin-stimulated cAMP reduction by ONO-5430608 at WT S1P$_5$ and mutants, measured by BRET-based EPAC sensor. Each data point represents mean ± SD for n = 3 independent experiments performed in triplicate. **i** Cell surface expression of the HA-tagged WT S1P$_5$ and mutants as determined by ELISA. Bar heights represent values ± SD for n = 3 independent experiments performed in triplicate. Source data are provided as a Source data file.

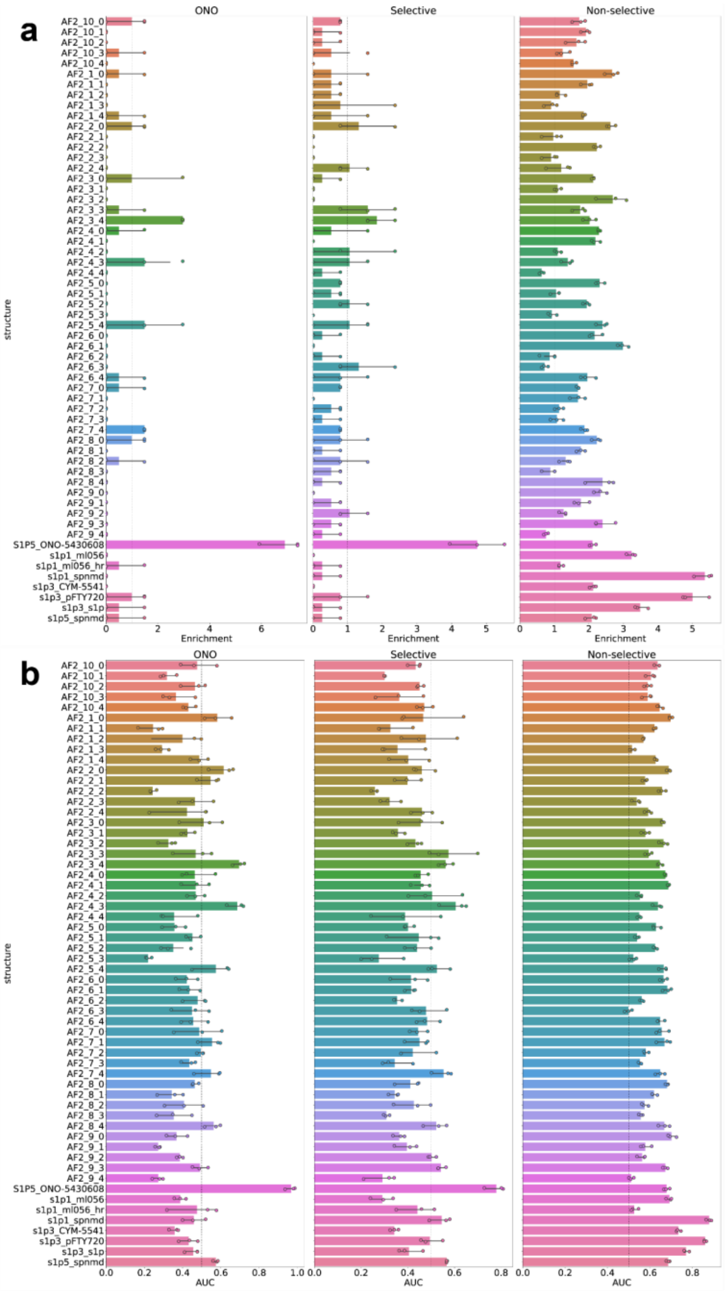

Supplementary Fig. 7 Virtual ligand screening benchmark comparison of AlphaFold models and experimental structures. **a** 10%-enrichment score, and **b** ROC-AUC score for 50 S1PR AlphaFold models and available experimental structures in three different benchmark sets. Bar heights represent mean ± 95% CI for n = 3 docking trials with effort = 1. Benchmark sets from left to right: 'ONO' – ligands from ONO-5430608 series[1], 'Selective' – selective $S1P_5$ ligands[1,2], 'Non-selective' - S1PR ligands (from ChEMBL[3]). Experimental structures used for screening: S1P5_ONO-5430608 (this work, PDB ID 7YXA), s1p1_ml056 (PDB ID 3V2W), s1p1_ml056_hr (PDB ID 3V2Y), s1p1-spnmd (PDB ID 7EVY), s1p3_CYM-5541 (PDB ID 7EW4), s1p3_pFTY720 (PDB ID 7EW2), s1p3-s1p (PDB ID 7C4S), s1p5-spnmd (PDB ID 7EW1).

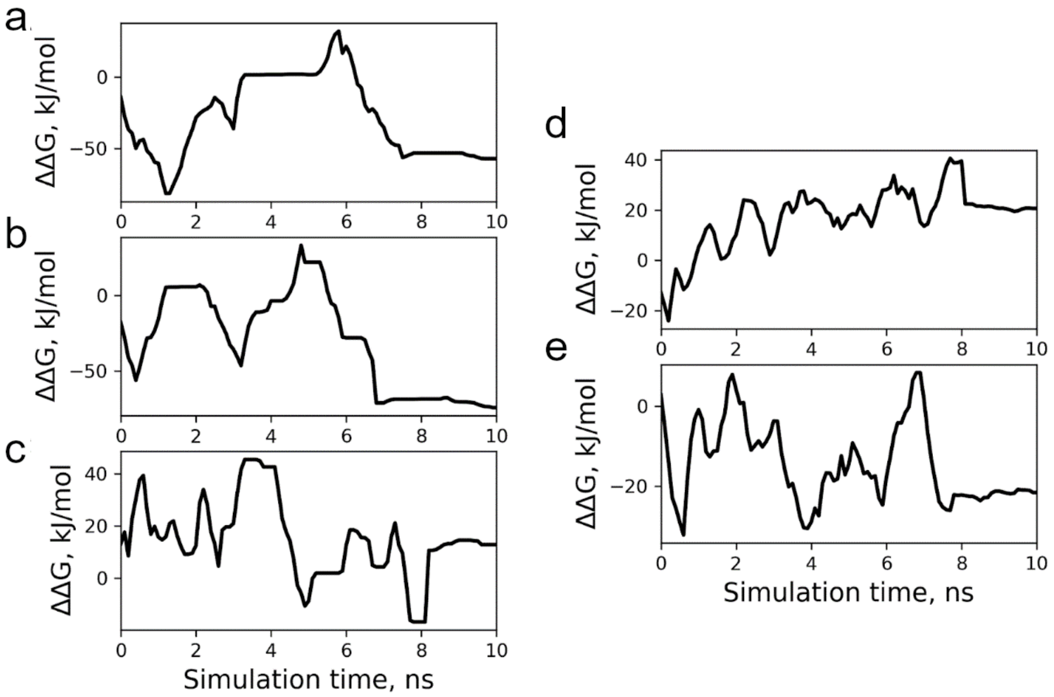

Supplementary Fig. 8 Convergence of metaMD simulations. a-c Convergence of metaMD free energy profiles of the $Y^{2.57}$ side chain torsion angle χ_1 in $S1P_1$ (**a**), $S1P_3$ (**b**), and $S1P_5$ (**c**). **d, e** Convergence of metaMD free energy profiles of the $L^{3.36}$ side chain torsion angle χ_1 in $S1P_5$ with upward (**d**) and downward (**e**) oriented $Y^{2.57}$. The convergence was estimated by tracking the difference between two distinct regions of the free energy profiles (ΔΔG) corresponding to the orientations of $Y^{2.57}$ in the $S1P_1$, $S1P_3$, and $S1P_5$ structures in (**a-c**) and to the orientations of $L^{3.36}$ in the active and inactive $S1P_5$ structures (**d, e**) as a function of simulation time. In case of convergence, this difference should not change with the progress of simulations as the systems diffuse freely along the reaction coordinate as observed during the last 2 ns of simulations on average.

Supplementary Tables

Supplementary Table 1 Crystallographic data collection and refinement statistics.

PDB ID	**$S1P_5$-ONO5430608 7YXA (XFEL)**	
**Data collection**		
Number of frames/crystals	6,818/7,492	
Space group	$P\ 2_1 2_1 2_1$	
Cell dimensions		
a, *b*, *c* (Å)	59.8, 103.4, 187.9	
α, β, γ (°)	90, 90, 90	
Resolution (Å)	30–2.2 (2.28–2.20)	
No. total reflections	7,236,474	
No. unique reflections	60,645	
R_{split} (%)	24.4 (71.8)	
Mean $I/\sigma I$	3.8 (1.5)	
Completeness (%)	100.0 (100.0)	
Multiplicity	119.3 (112.0)	
CC* (%)	97.9 (51.6)	
**Refinement**		
No. reflections/test set	60,071 (1,999)	
R_{work}/R_{free} (%)	30.07/32.92	
No. atoms		
Protein	2,804	2,808
Ligand	30	30
Water	194	
Lipid and other[b]	66	
Wilson *B*-factors ($Å^2$)	26.6	
Overall mean *B*-factors ($Å^2$)	A	B
$S1P_5$	36.6	45.8
BRIL	57.3	53.3
Ligand	34.6	32.3
Water	40.8	
Lipid and other[b]	49.5	
R.m.s. deviations		
Bond lengths (Å)	0.003	
Bond angles (°)	0.56	
Ramachandran stats (%)[c]		
Favored	97.13	
Allowed	2.87	
Outliers	0	

[a]Values in parentheses are for highest-resolution shell.
[b]NAG molecule bound to $N20^{N\text{-term}}$ in both chains is accounted in “other”.
[c]As defined by Molprobity[4]

Supplementary Table 2 Potencies (pEC_{50}) of S1P and ONO-5430608 at $S1P_5$ WT and mutants measured by BRET-based cAMP assay and cell surface expression.

Mutation	S1P, pEC_{50}, M	ONO, pEC_{50}, M	Surface Expression, %WT
WT	7.60 ± 0.13	8.77 ± 0.12	100 ± 8
$Y19^{NT}A$	N/R	N/R	86 ± 17
$Y19^{NT}F$	N/R	N/R	150 ± 30
$K24^{NT}A$	N/R	N/R	70 ± 20
$K24^{NT}Q$	N/R	N/R	150 ± 110
$K24^{NT}R$	7.3 ± 0.3	8.34 ± 0.08	150 ± 50
$C43^{1.39}F$	7.3 ± 0.1	8.35 ± 0.09	135 ± 6
$T79^{2.47}A$	6.71 ± 0.12	8.29 ± 0.14	98 ± 8
$Y89^{2.57}A$	N/R	N/R	83 ± 13
$Y89^{2.57}W$	6.99 ± 0.16	8.53 ± 0.19	44 ± 8
$N92^{2.60}A$	6.33 ± 0.05	N/R	78 ± 8
$N92^{2.60}C$	6.6 ± 0.3	N/R	145 ± 18
$R111^{3.28}A$	6.25 ± 0.03	N/R	140 ± 20
$R111^{3.28}Q$	5.58 ± 0.04	N/R	100 ± 50
$E112^{3.29}A$	5.83 ± 0.03	N/R	130 ± 60
$E112^{3.29}Q$	5.64 ± 0.04	N/R	100 ± 30
$V115^{3.32}A$	6.46 ± 0.17	7.53 ± 0.11	120 ± 30
$V115^{3.32}L$	6.4 ± 0.5	7.6 ± 0.2	140 ± 70
$V115^{3.32}M$	N/A	N/A	17 ± 10
$L292^{7.39}A$	6.9 ± 0.05	7.66 ± 0.12	152 ± 20
$L292^{7.39}V$	7.26 ± 0.09	7.63 ± 0.06	138 ± 18
$G293^{7.40}A$	6.23 ± 0.07	8.34 ± 0.07	120 ± 20
$G293^{7.40}V$	N/R	N/R	28 ± 8
$M296^{7.43}V$	N/R	N/R	52 ± 10
$M296^{7.43}W$	N/R	N/R	120 ± 20
$L318^{8.55}Q$	6.66 ± 0.05	7.65 ± 0.06	88 ± 18

Data represent mean ± SD of n = 3 independent experiments performed in triplicate.

N/R – no response.

N/A – data not available because of a low surface expression.

Supplementary Table 3 DNA sequence of the $S1P_5$ crystallization construct.

```
ATGAAGACCATCATCGCTCTGTCCTACATCTTCTGCCTCGTGTTCGCCgactacaaggacgatgacgatgctg
ggcgcgccatggaatcgggactcttgcgtccggctcctgtctctgaggtcatcgttctgcattacaactacactggaaaactgaggggtgcgaggt
accagcctggagctggattgagagctgacgcagtggtctgcctggcagtttgtgcgttcatcgtgctcgagaacttggctgtgctgctcgtcctggg
aaggcacccaagattccatgctccgatgttcttgctgctcggttcactcaccttgagtgatttgctggctggcgctgcctacgcagcgaacatcctctt
gtcgggaccactgaccctcaagttgtccccggctctgtggttcgccagagagggtggcgtcttcgttgctctgactgccagcgtcctctctctgctcg
caattgcgttggaacgctccctgacaatggcacgccgtggaccagcaccggtgtccagcagaggacgtacgctcgctatggctgcagcagcat
ggggagtctcattgctgctcggtttgctgccagctctgggatggaactgcctgggaagactcgacgcctgttccactgttctgccgctctacgctaag
gcctacgttctcttctgcgtgttggccttcgtcggcatcctcgctgccatttgcgcattgtacgcgaggatctactgtcaggtgagagcaaacgctgct
gatctggaagacaattgggaaactctgaacgacaatctcaaggtgatcgagaaggctgacaatgctgcacaagtcaaagacgctctgaccaa
gatgagggcagcagccctggacgctcagaaggccactccacctaagctcgaggacaagagcccagatagccctgaaatgaaagactttcg
gcatggattcgacattctggtgggacagattgatgatgcactcaagctggccaatgaagggaaagtcaaggaagcacaagcagccgctgagc
agctgaagaccacccggaatgcatacattcagaagtacctgcgccgtaaaccacgtagcctggctctcttgaggacactctctgttgtgctgctcg
ctttcgtcgcctgctggggaccactgttcttgctgctcttgctggacgtcgcatgccctgcgcgtacgtgtcccgttctcttgcaagccgatcctttcttgg
gtctggctatggccaactctctgctcaaccccatcatttacacattgacgaaccgcgacctgcgtcacgctttgctgaggctggtgggaagacctC
TGGAGGTGCTCTTCCAGGGTCCCCACCATCATCACCATCATCACCACCACCACTAA
```

Sequences of the cleaved tags and the stop codon are shown in capital letters.

Supplementary Table 4 Optimization of CrystFEL parameters and data quality improvement.

		A	B	C	C'	D	E	F	G	H	I
**Peak search**	SNR	4	2.7	4	4	4	4	4	4	4	4
	Threshold	100	30	30	30	30	30	30	30	30	30
	median-filter	-	-	5	5	5	5	5	5	5	5
**Integration**	regime	rings-nograd	rings-nograd	rings-nograd	rings-nograd	rings-grad	rings-grad	rings-grad	rings-grad	rings-grad	rings-grad
	int-radius	4,5,8	4,5,8	4,5,8	4,5,8	4,5,8	4,5,8	3,5,8	3,5,8	3,5,8	3,5,8
	local-bg-radius	-	-	-	-	-	5	5	5	5	5
**merging**	model	unity	unity	unity	xsphere	unity	unity	unity	ggpm	xsphere	xsphere
	pushres	3.0	3.0	3.0	2.0	3.0	3.0	3.0	5.0	1.5	2.0
	overpredict	-	-	-	-	-	-	-	-	-	yes
**Nframes**		1960	5007	6605	6582	6605	6594	6600	6918	7342	7348
**Ncrystals**		2036	5275	7189	7166	7185	7200	7205	7315	7787	7795
**Resolution**	low	30-5.4	30-5.4	30-5.4	30-5.4	30-5.4	30-5.4	30-5.4	30-5.4	30-5.4	30-5.4
	high	2.59-2.50	2.59-2.50	2.59-2.50	2.59-2.50	2.59-2.50	2.59-2.50	2.59-2.50	2.59-2.50	2.59-2.50	2.59-2.50
	overall	30-2.50	30-2.50	30-2.50	30-2.5	30-2.50	30-2.50	30-2.50	30-2.50	30-2.5	30-2.5
**Rsplit**	low	42.3	33.7	29.7	39.3	19.4	19.1	20.6	21.7	22.9	26.4
	high	241.3	206.5	186.0	214.2	61.7	60.0	48.1	49.6	78.5	72.8
	overall	57.0	48.9	44.7	55.4	23.8	23.6	21.1	21.8	27.3	28.6
**CC***	low	0.967	0.975	0.979	0.972	0.981	0.980	0.978	0.969	0.980	0.963
	high	0.554	0.587	0.618	0.582	0.666	0.676	0.716	0.712	0.688	0.679
	overall	0.964	0.975	0.978	0.968	0.984	0.983	0.983	0.978	0.982	0.973
**Redundancy**	low	96.8	314.1	503.8	167.2	149.8	148.9	124.9	113.3	55.0	44.4
	high	34.0	112.0	182.6	58.1	153.7	153.7	149.8	121.4	45.6	49.5
	overall	49.1	160.6	259.8	84.5	156.3	156.0	143.2	121.0	52.6	53.2
**I/sigma**	low	2.32	2.83	3.19	2.50	6.73	6.76	7.29	7.24	5.94	5.44
	high	0.47	0.52	0.58	0.51	1.87	1.88	2.41	2.23	1.41	1.54
	overall	1.38	1.61	1.78	1.52	4.12	4.12	4.97	4.79	3.54	3.55

Supplementary Table 5 Primers used in this study

Primer Name	Forward Primer (5'-to-3')	Reverse Primer (5'-to-3')
$S1P_5$ crystallization construct		
M1-V321	GATGCTGGGCGCGCCATGGAATCG GGACTCTTGCG	GCACCTCCAGAGGTCTTCCCACCAG CCTCAGCAAAGCG
A223-Bril-R241	GTCAGGTGAGAGCAAACGCTGCTG ATCTGGAAGACAATTGGG	GGCTACGTGGTTTACGGCGCAGGTA CTTCTGAATGTATGCATTCC
Functional tests primers		
L292A	CTC CTG CAG GCC GAT CCC TTC GCC GGA CTG GCC ATG GCC AAC TC	GAG TTG GCC ATG GCC AGT CCG GCG AAG GGA TCG GCC TGC AGG AG
Y19A	CGAGGTCATCGTCCTGCATgccAAC TACACCGGCAAGCTCC	GGAGCTTGCCGGTGTAGTTggcATGC AGGACGATGACCTCG
Y19F	CGAGGTCATCGTCCTGCATttcAACT ACACCGGCAAGCTCC	GGAGCTTGCCGGTGTAGTTgaaATGC AGGACGATGACCTCG
K24A	CTGCATTACAACTACACCGGCgccCT CCGCGGTGCGCG	CGCGCACCGCGGAGggcGCCGGTGT AGTTGTAATGCAG
K24Q	CTGCATTACAACTACACCGGCcagC TCCGCGGTGCGCG	CGCGCACCGCGGAGctgGCCGGTGT AGTTGTAATGCAG
K24R	CTGCATTACAACTACACCGGCcggC TCCGCGGTGCGCG	CGCGCACCGCGGAGccgGCCGGTGT AGTTGTAATGCAG
C43F	GCCGACGCCGTGGTGttcCTGGCGG TGTGCGCC	GGCGCACACCGCCAGgaaCACCACG GCGTCGGC
M296W	CCTTCCTGGGACTGGCCtggGCCAA CTCACTTCTGAACCCC	GGGGTTCAGAAGTGAGTTGGCccaG GCCAGTCCCAGGAAGG
M296V	CCTTCCTGGGACTGGCCgtgGCCAA CTCACTTCTGAACCCC	GGGGTTCAGAAGTGAGTTGGCcacG GCCAGTCCCAGGAAGG
Y89A	GGCAGGCGCCGCCgccGCCGCCAA CATCCTACTGTC	GACAGTAGGATGTTGGCGGCggcGG CGGCGCCTGCC
Y89W	GGCAGGCGCCGCCtggGCCGCCAA CATCCTACTGTC	GACAGTAGGATGTTGGCGGCccaGG CGGCGCCTGCC
N92A	CCGCCTACGCCGCCgccATCCTACT GTCGGGGCCG	CGGCCCCGACAGTAGGATggcGGCG GCGTAGGCGG

N92C	CCGCCTACGCCGCCtgcATCCTACT GTCGGGGCCG	CGGCCCCGACAGTAGGATgcaGGCG GCGTAGGCGG
E112A	CGCTCTGGTTCGCACGGgccGGAGG CGTCTTCGTGGC	GCCACGAAGACGCCTCCggcCCGTG CGAACCAGAGCG
E112Q	CGCTCTGGTTCGCACGGcagGGAG GCGTCTTCGTGGC	GCCACGAAGACGCCTCCctgCCGTGC GAACCAGAGCG
V115M	GCACGGGAGGGAGGCatgTTCGTGG CACTCACTGCG	CGCAGTGAGTGCCACGAAcatGCCTC CCTCCCGTGC
V115L	GCACGGGAGGGAGGCctgTTCGTGG CACTCACTGCG	CGCAGTGAGTGCCACGAAcagGCCT CCCTCCCGTGC
V115A	GCACGGGAGGGAGGCgccTTCGTG GCACTCACTGCG	CGCAGTGAGTGCCACGAAggcGCCT CCCTCCCGTGC
L318Q	TGCGCCACGCGCTCcagCGCCTGGT CTGCTGCG	CGCAGCAGACCAGGCGctgGAGCGC GTGGCGCA
R111Q	CGCGCTCTGGTTCGCAcagGAGGGA GGCGTCTTCGTG	CACGAAGACGCCTCCCTCctgTGCGA ACCAGAGCGCG
R111A	CGCGCTCTGGTTCGCAgccGAGGGA GGCGTCTTCGTG	CACGAAGACGCCTCCCTCggcTGCGA ACCAGAGCGCG
G293A	CAGGCCGATCCCTTCCTGgccCTGG CCATGGCCAACTC	GAGTTGGCCATGGCCAGggcCAGGA AGGGATCGGCCTG
G293V	CAGGCCGATCCCTTCCTGgtgCTGG CCATGGCCAACTC	GAGTTGGCCATGGCCAGcacCAGGA AGGGATCGGCCTG
L292V	CTGCAGGCCGATCCCTTCgtgGGAC TGGCCATGGCCA	TGGCCATGGCCAGTCCcacGAAGGG ATCGGCCTGCAG

Mutated codons are shown in lowercase.

Supplementary References

1. Watanabe, T., Kusumi, K. & Yuichil Inagaki, I. (2017). Tetrahydronaphthalene Derivative. United States patent No. US 2019/0031605A1 U.S. Patent and Trademark Office https://patentscope.wipo.int/search/en/detail.jsf?docId=US236791513
2. Ma, B. *et al.* Novel Potent Selective Orally Active S1P5 Receptor Antagonists. *ACS Med. Chem. Lett.* **12**, 351–355 (2021).
3. Davies, M. *et al.* ChEMBL web services: streamlining access to drug discovery data and utilities. *Nucleic Acids Res.* **43**, W612-W620 (2015).
4. Chen, V. B. *et al.* MolProbity: All-atom structure validation for macromolecular crystallography. *Acta Crystallogr. D Biol. Crystallogr.* **66**, 12-21 (2010).
