# Supplementary material for: Structural basis for receptor selectivity and inverse agonism in S1P5 receptors
Source: Nat Commun. 2022 Aug 12;13:4736. doi: 10.1038/s41467-022-32447-1 (PMC9374744; doi:10.1038/s41467-022-32447-1)
Supplement: Supplementary file 3 — Description of Additional Supplementary Files [file 41467_2022_32447_MOESM3_ESM.pdf]

File name: Supplementary Data 1

Description: The data file contains AlphaFold structures of S1P receptors, their sequences, and scripts used to generate them.

File name: Supplementary Data 2

Description: Supplementary Data file 2 includes structures of compounds used for docking to experimental and AlphaFold structures and their docking scores.
