# Supplementary material for: Structural basis for receptor selectivity and inverse agonism in S1P5 receptors
Source: Nat Commun. 2022 Aug 12;13:4736. doi: 10.1038/s41467-022-32447-1 (PMC9374744; doi:10.1038/s41467-022-32447-1)
Supplement: Supplementary file 6 — Reporting Summary [file 41467_2022_32447_MOESM6_ESM.pdf]

nature portfolio

Corresponding author(s): Cherezov V, Mishin A, Borshchevskiy V

Last updated by author(s): Jul 17, 2022

Reporting Summary

Nature Portfolio wishes to improve the reproducibility of the work that we publish. This form provides structure for consistency and transparency in reporting. For further information on Nature Portfolio policies, see our Editorial Policies and the Editorial Policy Checklist.

Statistics

For all statistical analyses, confirm that the following items are present in the figure legend, table legend, main text, or Methods section.

n/a	Confirmed	
☐	☒	The exact sample size (*n*) for each experimental group/condition, given as a discrete number and unit of measurement
☐	☒	A statement on whether measurements were taken from distinct samples or whether the same sample was measured repeatedly
☐	☒	The statistical test(s) used AND whether they are one- or two-sided<br>*Only common tests should be described solely by name; describe more complex techniques in the Methods section.*
☒	☐	A description of all covariates tested
☒	☐	A description of any assumptions or corrections, such as tests of normality and adjustment for multiple comparisons
☐	☒	A full description of the statistical parameters including central tendency (e.g. means) or other basic estimates (e.g. regression coefficient) AND variation (e.g. standard deviation) or associated estimates of uncertainty (e.g. confidence intervals)
☐	☒	For null hypothesis testing, the test statistic (e.g. *F*, *t*, *r*) with confidence intervals, effect sizes, degrees of freedom and *P* value noted<br>*Give P values as exact values whenever suitable.*
☒	☐	For Bayesian analysis, information on the choice of priors and Markov chain Monte Carlo settings
☒	☐	For hierarchical and complex designs, identification of the appropriate level for tests and full reporting of outcomes
☒	☐	Estimates of effect sizes (e.g. Cohen's *d*, Pearson's *r*), indicating how they were calculated

Our web collection on statistics for biologists contains articles on many of the points above.

Software and code

Policy information about availability of computer code

Data collection	Crystallographic data at SLS were collected using DA+ data acquisition and analysis software (http://scripts.iucr.org/cgi-bin/paper?S1600577517014503, version as provided on 24 February 2019)<br>Crystallographic data collection at PAL XFEL was on-line monitored with PAL XFEL NCI's OnDA (https://journals.iucr.org/j/issues/2016/03/00/zf5001/) and pre-processed using Cheetah v.2019-1.
Data analysis	CHARMM-GUI web-server (http://www.charmm-gui.org/; queried in 2021)<br>GRADE v.1.2.19 (http://grade.globalphasing.org; queried in November 2019)<br>Phenix v.1.19.2, WinCoot v.0.9.6, CrystFEL v.0.8.0, AlphaFold v.2.1.1+110948, Rotor-Gene Q v.2.3.1.49, GraphPad Prism v.9.3, Python v.3.7.4, Matplotlib v.3.3.2, Seaborn v.0.11.1, ICM Pro v.3.9-1b, Gromacs v.2020.2, RDKIT v.2021-03-4, Modeller v.9.24, PyMol v.2.4.1

For manuscripts utilizing custom algorithms or software that are central to the research but not yet described in published literature, software must be made available to editors and reviewers. We strongly encourage code deposition in a community repository (e.g. GitHub). See the Nature Portfolio guidelines for submitting code & software for further information.

Data

Policy information about availability of data

All manuscripts must include a data availability statement. This statement should provide the following information, where applicable:

- Accession codes, unique identifiers, or web links for publicly available datasets
- A description of any restrictions on data availability
- For clinical datasets or third party data, please ensure that the statement adheres to our policy

Coordinates and structure factors for the S1P5-ONO-5430608 structure have been deposited in the Protein Data Bank (PDB) under the accession code 7YXA

[https://doi.org/10.2210/pdb7YXA/pdb]. Raw SFX diffraction data have been deposited to CXIDB database under the accession number 196 [https://www.cxidb.org/id-196.html]. Publicly available amino acid sequences for S1PRs used in this study were obtained from the UniProt database under accession numbers: P21453 [https://www.uniprot.org/uniprot/P21453], O95136 [https://www.uniprot.org/uniprot/O95136], Q99500 [https://www.uniprot.org/uniprot/Q99500], O95977 [https://www.uniprot.org/uniprot/O95977], Q9H228 [https://www.uniprot.org/uniprot/Q9H228]. Publicly available structures used in this study can be found in the Protein Data Bank under accession codes: 3V2W [https://doi.org/10.2210/pdb3V2W/pdb], 3V2Y [https://doi.org/10.2210/pdb3V2Y/pdb], 4EIY [https://doi.org/10.2210/pdb4EIY/pdb], 7C4S [https://doi.org/10.2210/pdb7C4S/pdb], 7EVY [https://doi.org/10.2210/pdb7EVY/pdb], 7EW1 [https://doi.org/10.2210/pdb7EW1/pdb], 7EW2 [https://doi.org/10.2210/pdb7EW2/pdb], 7EW4 [https://doi.org/10.2210/pdb7EW4/pdb]. SNV data for S1P5 used in this work are available from public databases gnomAD [https://gnomad.broadinstitute.org/gene/ENSG00000180739?dataset=gnomad_r2_1] and COSMIC [https://cancer.sanger.ac.uk/cosmic/gene/analysis?ln=S1PR5]. AlphaFold structures, sequences, and scripts used to generate them are provided as a Supplementary Data file 1. Structures of the compounds used for docking to experimental and AlphaFold structures and their docking scores are provided as a Supplementary Data file 2. Source data are provided with this paper.

Field-specific reporting

Please select the one below that is the best fit for your research. If you are not sure, read the appropriate sections before making your selection.

- [x] Life sciences
- [ ] Behavioural & social sciences
- [ ] Ecological, evolutionary & environmental sciences

For a reference copy of the document with all sections, see nature.com/documents/nr-reporting-summary-flat.pdf

Life sciences study design

All studies must disclose on these points even when the disclosure is negative.

Sample size	No statistical methods were used to predetermine sample size. BRET-based cAMP signaling assays were conducted at least in n=3 biologically independent experiments and are comparable to other published studies (Salahpour et al. 2012 Front Endocrinol. 3: 105; Espinoza et al. 2012 Methods Mol. Biol. 964:107-22; Aleksandrov et al. 2018 Neurotox. Res. 34: 442-451; Maguire et al 2012 Methods Mol. Biol. 897, 31-77). Serial femtosecond crystallography data were collected from 7,492 crystals to ensure at least 100-fold multiplicity in all resolution shells.
Data exclusions	No data were excluded.
Replication	For BRET-based cAMP assays, three biologically independent experiments were performed in triplicate. All attempts at replication were successful. Crystallization trials have been successfully repeated at least 4 times with reproducibly diffracting crystals.
Randomization	This study did not allocate samples in experimental groups thus no randomization was required for the reported experiments.
Blinding	The researchers were not blinded to allocation during experiments and outcome assessment. Blinding was not required for the reported experiments because all functional and structural data were analyzed using the same methods, and results are not subjective.

Reporting for specific materials, systems and methods

We require information from authors about some types of materials, experimental systems and methods used in many studies. Here, indicate whether each material, system or method listed is relevant to your study. If you are not sure if a list item applies to your research, read the appropriate section before selecting a response.

Materials & experimental systems

n/a	Involved in the study	
[ ]	[x]	Antibodies
[ ]	[x]	Eukaryotic cell lines
[x]	[ ]	Palaeontology and archaeology
[x]	[ ]	Animals and other organisms
[x]	[ ]	Human research participants
[x]	[ ]	Clinical data
[x]	[ ]	Dual use research of concern

Methods

n/a	Involved in the study	
[x]	[ ]	ChIP-seq
[x]	[ ]	Flow cytometry
[x]	[ ]	MRI-based neuroimaging

Antibodies

Antibodies used	Rat monoclonal anti-HA tag antibody coupled to HRP (clone 3F10, Roche Cat# 12013819001)
Validation	Anti-HA-Peroxidase, High Affinity antibody (3F10) recognizes the HA peptide sequence [YPYDVPDYA] derived from the influenza hemagglutinin protein (Kolodziej and Young, 1991, Meth Enzymol 194, 508-511). The Anti-HA-Peroxidase, High Affinity (3F10) antibody is function tested by Western blot analysis using a cell line, that expresses a recombinant HA-tagged protein.

Eukaryotic cell lines

Policy information about cell lines

Cell line source(s)	Sf9 cells were purchased from MilliporeSigma (Novagen, cat 71104), HEK293T cells were purchased from European Collection of Authenticated Cell Cultures (ECACC, cat 12022001)
Authentication	The cell lines were authenticated by the supplier (Novagen, ECACC) using morphology and growth characteristics
Mycoplasma contamination	Both Sf9 and HEK293T cells have been tested and shown to be free from mycoplasma
Commonly misidentified lines (See ICLAC register)	No commonly misidentified cell line were used
